# TTF-1 negativity in synchronous M1b/M1c wildtype lung adenocarcinoma brain metastases predicts worse survival with increased risk of intracranial progression

**DOI:** 10.1007/s11060-024-04885-y

**Published:** 2024-12-04

**Authors:** David Wasilewski, Tommaso Araceli, Philip Bischoff, Anton Früh, Rober Ates, Selin Murad, Niklas Jung, Jan Bukatz, Majd Samman, Katharina Faust, Julia Jünger, Martin Witzenrath, David Horst, Atik Baborie, Arend Koch, David Capper, Frank L. Heppner, Helena Radbruch, Markus J. Riemenschneider, Nils Ole Schmidt, Peter Vajkoczy, Martin Proescholdt, Julia Onken, Nikolaj Frost

**Affiliations:** 1 Department of Neurosurgery, Charité–Universitätsmedizin Berlin, Corporate Member of Freie Universität Berlin and Humboldt-Universität Zu Berlin, Berlin, Germany; 2 Charité Comprehensive Cancer Center, Charité–Universitätsmedizin Berlin, Corporate Member of Freie Universität Berlin and Humboldt-Universität Zu Berlin, Berlin, Germany; 3 German Cancer Consortium (DKTK), partner site Berlin, and German Cancer Research Center (DKFZ), Heidelberg, Germany; 4 Department of Neurosurgery, University Regensburg Medical Center, Regensburg, Germany; 5 Wilhelm-Sander Neuro-Oncology Unit, University Regensburg Medical Center, Regensburg, Germany; 6 Berlin Institute of Health at Charité–Universitätsmedizin Berlin, Berlin Institute of Health (BIH) Charité, Charitéplatz 1, 10117 Berlin, Germany; 7 Institute of Pathology, Charité–Universitätsmedizin Berlin, Corporate Member of Freie Universität Berlin and Humboldt-Universität Zu Berlin, Berlin, Germany; 8 Department of Neurosurgery, Neuroscience Institute, King Salman Medical City, Medina, Saudi Arabia; 9 Institute of Neuropathology, Charité–Universitätsmedizin Berlin, Corporate Member of Freie Universität Berlin and Humboldt-Universität Zu Berlin, Berlin, Germany; 10 Department of Infectious Diseases and Pulmonary Medicine, Charité–Universitätsmedizin Berlin, Corporate Member of Freie Universität Berlin and Humboldt-Universität Zu Berlin, Berlin, Germany; 11 Department of Neuropathology, University Regensburg Medical Center, Regensburg, Germany

**Keywords:** LUAD, Brain Metastasis, TTF-1, Ki67, Tumor volume, Edema volume, Survival

## Abstract

**Background:**

Thyroid Transcription Factor-1 (TTF-1) expression in lung adenocarcinoma (LUAD) has been studied for its prognostic value in early-stage and metastatic disease. Its role in brain metastasis remains unexplored. This study investigates the predictive value and association of TTF-1 status with clinicopathological variables in patients with synchronous LUAD brain metastases.

**Material and methods:**

In this bicentric retrospective study, 245 patients with newly diagnosed, treatment-naïve brain metastasis undergoing resection were included. Patient data were retrieved from electronic records. Outcomes included overall and progression-free survival. Statistical analysis included Kaplan–Meier estimates and Cox proportional hazards regression.

**Results:**

Mean Ki67 index in TTF-1 negative patients was 43% [95% CI 38–48%] compared to 32% [95% CI 29–35%] in TTF-1 positive (TTF-1 +) patients (p < 0.001). Tumor volume was significantly larger in TTF-1 negative (TTF-1-) patients (mean volume 24 mL [95% CI 18–31 mL]) vs. 15 mL [95% CI 12–17 mL] in TTF-1 + patients (padjust = 0.003). Perifocal edema was smaller in TTF-1- patients (mean volume: 58 mL [95% CI 45–70 mL]) vs. 84 mL [95% CI 73–94 mL] in TTF-1 + patients (padjust = 0.077). Tumor and edema volume did not correlate. TTF-1- patients showed worse overall, intracranial, and extracranial progression-free survival. In a multivariable Cox model, positive TTF-1 status was independently associated with improved outcomes. Negative TTF-1 status was associated with increased hazard for intracranial disease progression compared to extracranial progression.

**Conclusion:**

In synchronous LUAD brain metastases, TTF-1 negativity reflects an aggressive phenotype with larger proliferation capacity and tumor volume. Future research should explore the underlying cellular and molecular alterations of this phenotype.

**Supplementary Information:**

The online version contains supplementary material available at 10.1007/s11060-024-04885-y.

## Introduction

Brain metastases pose a significant clinical challenge in patients with non-small cell lung cancer (NSCLC), affecting up to 50% of patients during the disease [1]. Lung adenocarcinoma (LUAD) is the most common subtype of NSCLC leading to brain metastases. Traditional local treatments, such as radiation therapy, including stereotactic radiosurgery (SRS), and microsurgical resection by neurosurgeons, aim to achieve local intracranial disease control [1]. The landscape of systemic therapy of the primary tumor, extracranial metastatic disease as well as brain metastases has changed with the advent of targeted therapies including antibody-based therapies, small molecule inhibitors (SMIs) and immune checkpoint inhibition (ICI). In certain patients, these therapies demonstrated a profound impact on overall survival (OS) and progression-free survival (PFS) [1]. Thyroid transcription factor 1 (TTF-1), also known as NKX2-1, is a homeodomain transcription factor crucial in controlling gene expression in lung tissue and influencing biological processes such as cell differentiation and morphogenesis [2, 3]. TTF-1 is expressed in 60–90% of LUAD patients and thyroid carcinomas but is typically absent in adenocarcinomas of other sites and NSCLC squamous cell carcinomas as detected by immunohistochemistry (IHC) [3–6]. Clinically, TTF-1 serves as a diagnostic biomarker to differentiate LUAD from other adenocarcinoma metastases of extra-thoracic origin [4, 5]. There is substantial evidence demonstrating the prognostic and predictive value of TTF-1 in LUAD, with its expression associated with favorable prognosis, independent of tumor stage [7–13]. In a previously published propensity score matched-based analysis it was demonstrated that TTF-1 expression in LUAD was linked to improved OS and PFS [10]. Recently, in a bicentric cohort of patients with resected LUAD, TTF-1 positivity was associated with longer disease-free survival (DFS), irrespective of tumor grade, as shown by a Bayesian network analysis [11]. Brain metastases at initial diagnosis can affect up to 16% of patients with LUAD at initial diagnosis [13–15]. These patients with first-diagnosed, therapy-naïve LUAD and clinically detectable brain metastases may present without further extra-thoracic metastases corresponding to oligometastatic disease (M1b) or additional extra-thoracic metastases (M1c) [16]. Here data on the relationship between TTF-1 expression and clinicopathological characteristics of brain metastases and the predictive value of TTF-1 status are lacking. Similarly, it remains unclear as to whether TTF-1 status based on analysis of brain metastasis tissue can offer prognostic information. In this retrospective, bicentric study we evaluated a large cohort of patients with newly diagnosed synchronous LUAD brain metastases that underwent microsurgical resection of brain metastases and assessed the relationship between histopathological as well as anatomical parameters and TTF-1 status and the role of TTF-1 status in terms of predicting patient outcome.

## Methods

### Patient cohort and clinical data

This retrospective, bicentric observational study included 245 treatment-naïve patients with newly diagnosed, synchronous brain metastases from LUAD without EGFR/ALK driver mutations.

By German ethical and regulatory standards and the Declaration of Helsinki (7th revision, 2013), the study was approved by our Institutional Ethics Review Boards (votes no. EA1/399/20 and no. 20-1799-101). Patients were classified into M1b and M1c categories based on the UICC Stage IV (8th edition TNM classification). The M1b category denotes Stage IV disease with a single extrathoracic metastasis, in our study implying a solitary brain metastasis. In contrast, M1c represents Stage IV disease characterized by multiple extrathoracic metastases. All patients underwent brain metastasis resection between January 2010 and December 2023 at two high-volume university neurosurgical centers (Supplementary Fig. 1). Clinical data, including age, gender, post-operative Karnofsky Performance Score (KPS), and diagnosis-specific graded prognostic assessment (ds-GPA), were collected from electronic patient records. Anatomical and radiological data were derived from cranial MRI (cMRI) or computer tomography (CT) staging, noting the dominant brain metastasis site, number of metastases, extracranial metastases, hydrocephalus, and leptomeningeal disease (LMD). Following institutional standards consistent with methods used in other neurosurgical research groups [17, 18]. Tumor volumes and edema volumes were quantified using a semi-automated 3D rendering algorithm in iPlannet (Brainlab, Munich, Germany) with the SmartBrush tool. The volumetric measurements (3D) rendering was based on pre-operative planning by the operating neurosurgeon and were performed by three authors (D. W., J. B. and T. A).

Radiologic tumor response was evaluated using RECIST (version 1.1) and iRANO criteria based on retrospective chart reviews (CR, PR, SD, or PD). Progression-free survival (PFS) was categorized into extracranial PFS (ecPFS) and intracranial PFS (icPFS). Median follow-up was estimated using the reverse Kaplan–Meier method, with OS, icPFS, and ecPFS defined from the time of neurosurgical resection to death or last follow-up. Treatment-related characteristics were retrieved from electronic patient records and related to post-operative treatment. Patients were classified into those receiving no systemic therapy (i.e. best supportive care or radiation therapy only) vs. those receiving systemic therapy (i.e. radiation therapy and chemotherapy, radiation therapy and ICI and radiation therapy with targeted therapies, i.e. SMIs or antibody-based therapy (Table 1).

**Table 1:** Patient characteristics at baseline. Summary of baseline features of the study cohort (n = 245) of treatment-naïve, UICC stage IV NSCLC/ADC (LUAD) patients. All characteristics refer to the time of first brain metastasis resection (baseline), if not stated otherwise. KPS and ds-GPA were arbitrarily dichotomized into “good” and “bad”. Furthermore, clinical variables are listed according to TTF-1 status. Brain metastases to be resected (dominant brain metastases) were classified across various brain regions: frontal, parietal, temporal, occipital, cerebellar, and others (e.g. insular region, lesions associated with the third ventricle, lesions of the skull base. Unknown values are displayed as NA

Characteristic	N	Overall N = 245	TTF-1- N = 75 (95% CI)^*1*^	TTF-1 + N = 170 (95% CI)^*1*^	p-value^*2*^	q-value^*3*^
Age, Mean	245	64	65 (62, 67)	64 (62, 66)	0.42	0.55
Gender, n (%)	245				0.17	0.40
Female		101 (41)	26 (24%, 47%)	75 (37%, 52%)		
Male		144 (59)	49 (53%, 76%)	95 (48%, 63%)		
Post-operative KPS, n (%)	245				0.39	0.55
KPS < 70% (“Bad KPS”)		85 (35)	29 (28%, 51%)	56 (26%, 41%)		
KPS ≥ 70% (“Good KPS”)		160 (65)	46 (49%, 72%)	114 (59%, 74%)		
ds-GPA score, n (%)	245				0.28	0.48
GPA ≤ 2 (“Bad GPA”)		151 (62)	50 (55%, 77%)	101 (52%, 67%)		
GPA > 2 (“Good GPA”)		94 (38)	25 (23%, 45%)	69 (33%, 48%)		
Affected anatomical region of the resected brain metastasis, n (%)	245				0.13	0.40
Cerebellar		63 (26)	23 (21%, 43%)	40 (18%, 31%)		
Frontal		88 (36)	22 (20%, 41%)	66 (32%, 47%)		
Occipital		24 (9.8)	5 (2.5%, 16%)	19 (7.0%, 17%)		
Other		6 (2.4)	1 (0.07%, 8.2%)	5 (1.1%, 7.1%)		
Parietal		42 (17)	19 (16%, 37%)	23 (8.9%, 20%)		
Temporal		22 (9.0)	5 (2.5%, 16%)	17 (6.1%, 16%)		
Brain Metastasis Location, n (%)	245				0.41	0.55
Infratentorial		38 (16)	15 (12%, 31%)	23 (8.9%, 20%)		
Supratentorial		145 (59)	41 (43%, 66%)	104 (53%, 68%)		
Both		62 (25)	19 (16%, 37%)	43 (19%, 33%)		
Tumor Volume (mL), Mean	235	18	24 (18, 31)	15 (12, 17)	< 0.001	0.003
NA		10	5	5		
Edema Volume (mL), Mean	199	76	58 (45, 70)	84 (73, 94)	0.018	0.077
NA		46	19	27		
Hydrocephalus at time of brain metastasis resection, n (%)	245				0.15	0.40
Hydrocephalus		34 (14)	14 (19) (11%, 30%)	20 (12) (7.5%, 18%)		
No hydrocephalus		211 (86)	61 (81) (70%, 89%)	150 (88) (82%, 92%)		
Brain Metastasis Burden, n (%)	245				0.90	> 0.99
1		90 (37)	26 (24%, 47%)	64 (30%, 45%)		
2		72 (29)	23 (21%, 43%)	49 (22%, 36%)		
> 2		83 (34)	26 (24%, 47%)	57 (27%, 41%)		
Leptomeningeal Disease, n (%)	245				> 0.99	> 0.99
LMD after brain metastasis resection		10 (4.1)	3 (1.0%, 12%)	7 (1.8%, 8.6%)		
No LMD after brain metastasis resection		235 (96)	72 (88%, 99%)	163 (91%, 98%)		
Extracranial Metastases, n (%)	245				0.23	0.48
No presence of extracranial metastases at baseline		154 (63)	43 (45%, 69%)	111 (58%, 72%)		
Presence of extracranial metastases at baseline		91 (37)	32 (31%, 55%)	59 (28%, 42%)		
PD-L1 TPS (Brain metastasis tissue), Mean	124	19	14 (4.6, 23)	21 (14, 27)	0.28	0.48
NA		121	40	81		
Ki67 Index (Brain metastasis tissue), Mean	204	35	43 (38, 48)	32 (29, 35)	< 0.001	0.001
Unknown		41	10	31		
Lung tumor resection, n (%)	245				0.95	> 0.99
No primary tumor resection		202 (82)	62 (72%, 90%)	140 (76%, 88%)		
Primary tumor resection before or after brain metastasis removal		43 (18)	13 (9.9%, 28%)	30 (12%, 24%)		
Postsurgical systemic treatment, n (%)	245				0.80	0.97
Best supportive care		22 (9.0)	8 (5.0%, 20%)	14 (4.7%, 14%)		
Radiation		41 (17)	13 (9.9%, 28%)	28 (11%, 23%)		
Radiation and chemotherapy		101 (41)	33 (33%, 56%)	68 (33%, 48%)		
Radiation and immunotherapy		68 (28)	17 (14%, 34%)	51 (23%, 38%)		
Radiation and targeted therapy		13 (5.3)	4 (5.3) (1.7%, 14%)	9 (5.3) (2.6%, 10%)		

^*1*^CI Confidence Interval

^*2*^Wilcoxon rank sum test; Pearson’s Chi-squared test; Fisher’s exact test

^*3*^False discovery rate correction for multiple testing

### Histopathological data

TTF-1 status was assessed based on the dominant (index) lesion. In cases where multiple tumors were resected (i.e. two-stage resections within a 14-day) the tissue of the first operation was used for analysis of TTF-1. TTF-1 status was determined by immunohistochemical staining of formalin-fixed, paraffin-embedded (FFPE) brain metastasis tissue using the 8G7G3/1 antibody (Dako, 1:100). A board-certified neuropathologist classified TTF-1 positivity as any nuclear staining in tumor cells (Supplementary Fig. 2). Additional testing for EGFR mutations, ALK, and ROS1 genes was conducted using NGS and PCR as part of standard clinical practice. PD-L1 tumor proportion score (TPS) was assessed via Dako-Agilent’s PD-L1 IHC 22C3 pharmDx or as a validated 22C3-test on Dako-Agilent Omni’s machine. As for Ki67 staining immunohistochemistry was performed on a Benchmark XT autostainer (Ventana Medical Systems) with standard antigen retrieval methods (CC1 buffer, pH8.0) using monoclonal mouse anti-MIB1 (Ki-67, 1:100, Dako M7240) [19].

### Statistical analysis

Statistical analyses were conducted using GraphPad Prism (v9, GraphPad Software, Inc., San Diego, USA) and R Studio (v2023.09.0 + 463, R Foundation for Statistical Computing, Inc., Boston, USA). Descriptive statistics summarized clinical, histopathological, radiological, and treatment data, with calculations performed in Excel and R. Continuous variables were compared using the Mann–Whitney U test, while categorical variables were compared using Fisher’s exact or Chi-square tests. The gtsummary R package (v0.4.3) was utilized to create the clinical data table. Kaplan–Meier analysis provided median intracranial progression-free survival (icPFS), extracranial progression-free survival (ecPFS), and overall survival (OS) estimates, with 95% confidence intervals (CIs) and differences assessed via log-rank tests, plotted using the survminer package. Multivariable Cox regression for OS, ecPFS, and icPFS included cases with complete data and relevant clinical covariates. To account for inter-patient variability, we employed a frailty model within the Cox proportional hazards framework, incorporating a gamma-distributed random effect for each patient. This approach captured unobserved heterogeneity and controlled for patient-specific baseline hazard variations, providing a robust assessment of TTF-1 status on progression risks. Additional analyses utilized R packages dplyr (v1.1.4), tidyverse (v2.0.0), and corrplot (v0.92). Statistical significance was set at p < 0.05, with p-values between > 0.05 and ≤ 0.1 considered trends. R code and raw data are available upon request.

## Results

### Patient characteristics

**Fig. 1:**
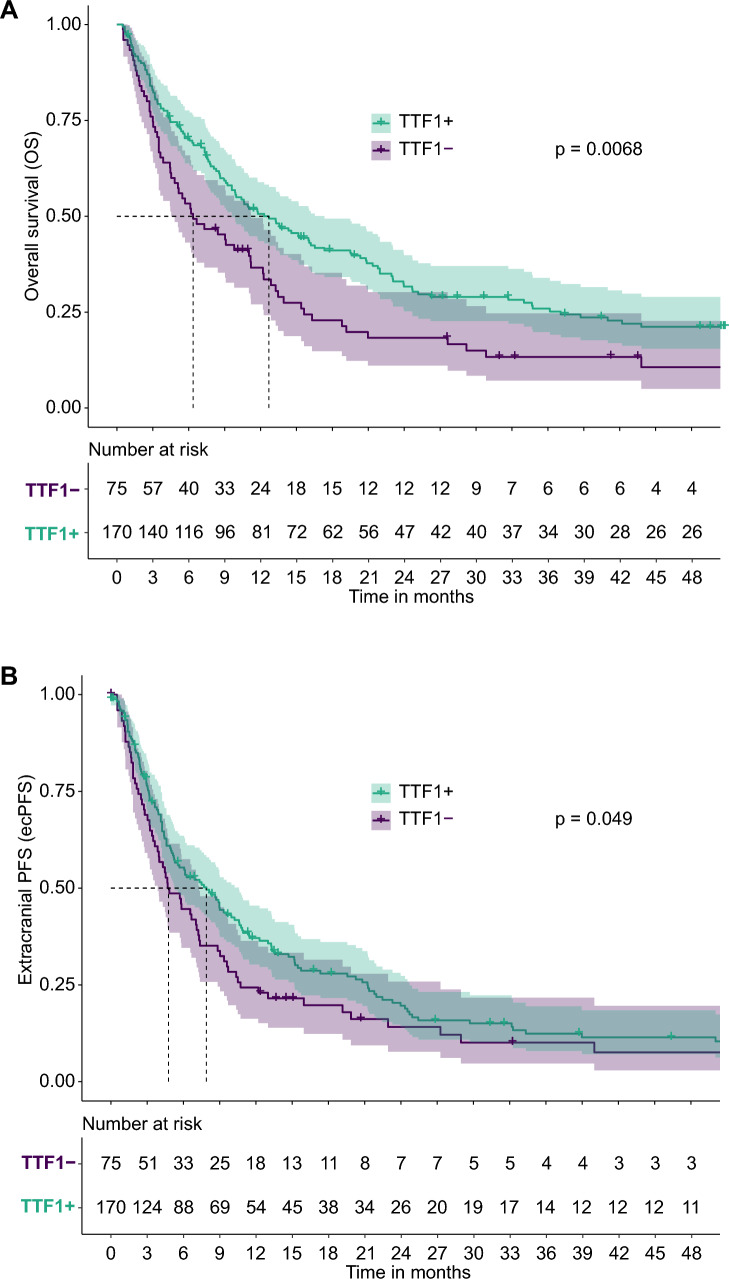

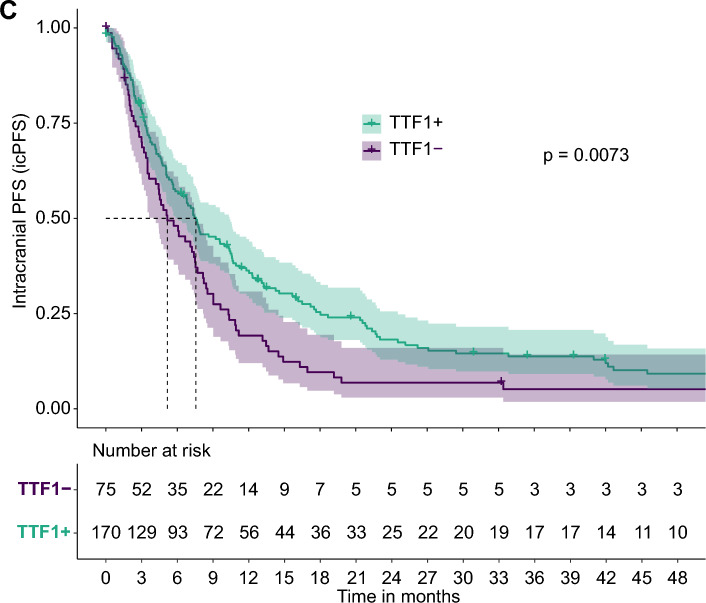
Kaplan–Meier estimates for OS, ecPFS and icPFS. Patients were dichotomized according to TTF-1 status (**A**–**C**). The survival curves are color-coded for clarity, with confidence intervals shaded lightly around the curves. Statistical significance is annotated on the graph where applicable

Patient selection is displayed in Supplementary Fig. 1 with baseline characteristics being summarized in Table 1. The median follow-up time from the first brain metastasis resection was 57.7 months [95% CI 50.5–73.0], while the median OS was 10.6 months [95% CI 8.6–13.3]. The median ecPFS was 6.8 months [95% CI 5.0–8.3] and the median icPFS was 7.9 months [95% CI 5.8–10.5] (Supplementary Fig. 2). 75 patients (31.0%) were TTF-1- and 170 patients (69.0%) TTF-1 + (Table 1). TTF-1 + patients had a median OS of 12.70 months [95% CI 9.60–17.3], while TTF-1- patients had a median OS of 6.37 months [95% CI 4.53–11.2]. As for ecPFS, TTF-1 + patients had a median ecPFS of 7.90 months [95% CI 5.30–10.6], whereas TTF-1- patients had a median ecPFS of 4.77 months [95% CI 3.87–7.3]. In terms of icPFS, TTF-1 + patients had a median icPFS of 7.57 months [95% CI 6.07–10.40], while TTF-1- patients had a median icPFS of 5.17 months [95% CI 4.20–7.53] (Fig. 1A–C). The most common locations of the index brain metastasis included the frontal (36.0%) or parietal lobe (17.0%) and cerebellum (26.0%). The mean tumor volume and volume of associated perifocal edema were 18 mL [95% CI 14–20] and 76 mL [95% CI 66–84], respectively (Table 1). No difference in the incidence of LMD occurrence during the follow-up of our patients between the TTF-1 + and TTF-1- group of patients was observed.

### Correlation of TTF-1 status with clinical and pathological characteristics

**Fig. 2:**
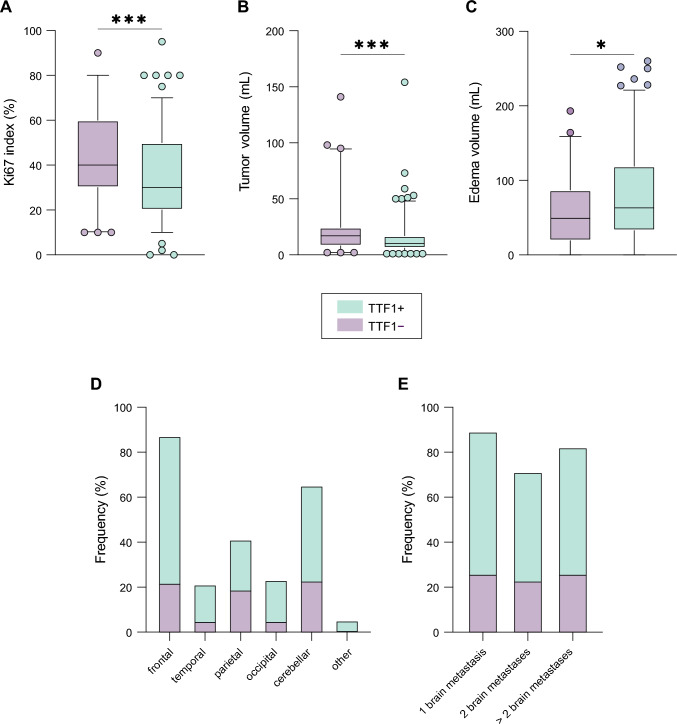
Relationship between TTF-1 status and anatomical parameters grouped according to TTF-1 status. Bar plot showing the relationship between Ki67 proliferation index and TTF-1 status (**A**), tumor volume (in mL) and TTF-1 status (**B**), and TTF-1 status and edema volume in milliliters (mL) (**C**), compared between TTF-1- and TTF-1 + patients. A grouped bar plot demonstrates frequencies of TTF-1- and TTF-1 + patients depending on the anatomical localization of the dominant brain metastasis (**D**). Brain metastasis burden (classified in three categories: “1 brain metastasis,” “2 brain metastases,” and “ > 2 brain metastases”) and frequency of TTF-1- and TTF-1 + patients are indicated by grouped bar plots (**E**). Two-tailed Mann–Whitney U tests were used for pairwise comparisons to calculate p-values

There was no significant correlation between edema and tumor volume, but TTF-1 status correlated significantly with tumor and edema volume (Supplementary Fig. 4). Additionally, TTF-1 status negatively correlated with tumor volume (Spearman’s correlation coefficient = − 0.39) and Ki67 index (correlation coefficient = − 0.09), and positively with edema volume (correlation coefficient = 0.22) (Supplementary Fig. 4). In TTF-1- patients, the mean tumor volume was 24 mL [95% CI 18–31 mL], compared to 15 mL [95% CI 12–17] in TTF-1 + patients (adjusted p-value (p_adjust_) = 0.003) (Table 1, Fig. 2A, Supplementary Fig. 3A). TTF-1- patients also had significantly lower edema volume at 58 mL [95% CI 45–70] versus 84 mL [95% CI 73–94] in TTF-1 + patients (p = 0.018) (Table 1, Fig. 2B, Supplementary Fig. 5), although the adjusted p-value was not significant (p_adjust_ = 0.077) (Table 1). Other variables, such as the location of the dominant brain metastasis, the incidence of hydrocephalus, and the intracranial brain metastasis burden, did not significantly differ between TTF-1- and TTF-1 + patients (Table 1, Fig. 2D, E). Interestingly, PD-L1 TPS in brain metastasis tissue was lower in TTF-1- patients than in TTF-1 + patients, although there was no significant difference (Table 1). Importantly, TTF-1- and TTF-1 + patients did not show any difference concerning pre-operative cumulative dexamethasone (p = 0.31) with no clear association of pre-operative cumulative dexamethasone doses with tumor and edema volume in TTF-1 + and TTF-1- patient groups (Supplementary Fig. 6).

### Association of TTF-1 status with patient outcomes

**Fig. 3:**
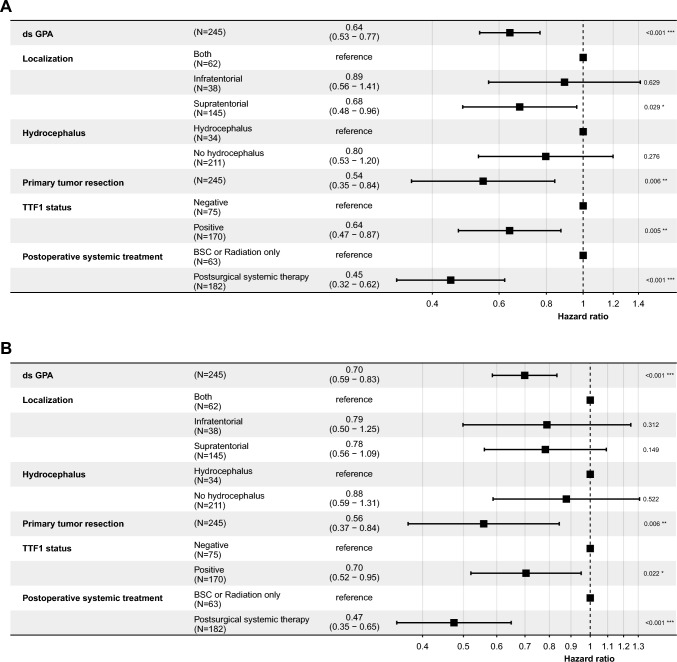

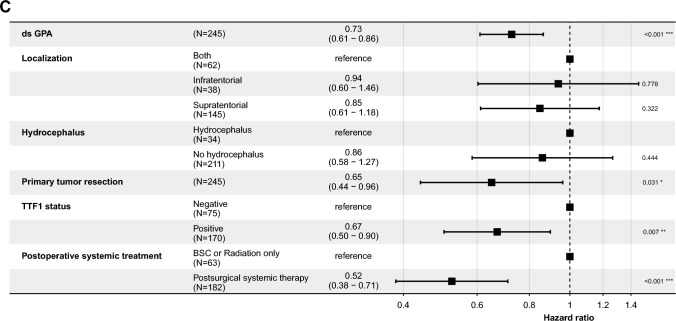
Multivariable Cox regression analysis for OS. Forest plots reporting hazard ratios and 95% confidence intervals for OS (**A**), ecPFS (**B**) and icPFS (**C**) of the total cohort of 245 patients. Forest plots reporting hazard ratios and 95% confidence intervals for OS. The vertical dashed line signifies a hazard ratio of 1.0, p-values indicated, and significant p-values highlighted in bold

**Fig. 4:**
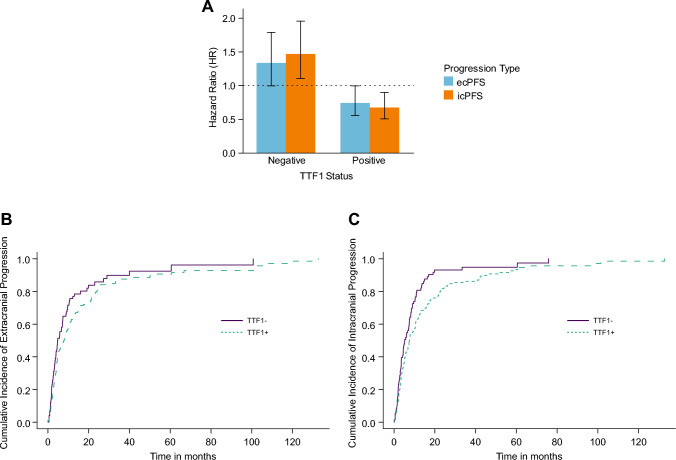
Hazard ratio for intracranial and extracranial progression-free survival and cumulative Incidence of ecPFS and icPFS by TTF-1 Status. This figure displays the hazard ratios (HRs) and 95% confidence intervals (CIs) for TTF-1 + and TTF-1- patients, stratified by ecPFS and icPFS. The hazard ratios are represented as bars, with error bars indicating the 95% confidence intervals. The colors distinguish between the progression types, with ecPFS shown in purple and icPFS in green. A dashed horizontal line at HR = 1 indicates no effect. This figure visualizes the relative risk reduction or increase for TTF-1 + and TTF-1- patients across the different progression types (**A**). The cumulative incidence functions (CIFs) for ecPFS (**B**) and for icPFS (**C**) stratified by TTF-1 status (TTF-1 + and TTF-1-). The plot includes the cumulative incidence estimates over time. Legends indicate the different TTF-1 status groups, with TTF-1- shown in purple and TTF-1 + in green

A positive TTF-1 status was associated with a significantly decreased hazard for death, with a hazard ratio (HR) of 0.64 [95% CI 0.47–0.87], indicating a 36% reduction in risk compared to the reference group (Fig. 3B p < 0.001). The hazard for extracranial disease progression was also lower in patients with a positive TTF-1 status, showing an HR of 0.70 [95% CI 0.52–0.95], translating to a 30% reduction in risk (Fig. 3B p = 0.022). For intracranial disease progression, the HR was 0.67 [95% CI 0.50–0.90], indicating a 33% reduction in risk (Fig. 3C p = 0.007). TTF-1 + patients exhibited a reduced risk for extracranial progression (HR of 0.75 [95% CI 0.56–1.0]) compared to TTF-1- patients (HR of 1.34 [95% CI 1.0–1.8]). For icPFS, TTF-1 + patients demonstrated a significantly reduced risk with an HR of 0.68 [95% CI 0.51–0.90], indicating statistical significance. TTF-1- patients had a higher risk with an HR of 1.48 [95% CI 1.1–2.0]. When comparing the ratios of intracranial to extracranial progression risks, TTF-1 + patients had a ratio of 0.91, suggesting a slightly lower relative risk of intracranial progression. Conversely, TTF-1- patients had a ratio of 1.10, indicating a higher relative risk of intracranial progression compared to extracranial progression (Fig. 4A–C). Additional factors analyzed included GPA status, primary tumor resection, and postsurgical systemic treatment. GPA status had an HR of 0.69 [95% CI 0.57–0.83] (p < 0.001), suggesting a significant association with survival. Primary tumor resection showed an HR of 0.74 [95% CI 0.58–0.95] (p = 0.017), while postsurgical systemic treatment was associated with an HR of 0.77 [95% CI 0.61–0.97] (p = 0.027).

## Discussion

Patients with treatment-naïve patients with LUAD and synchronous brain metastases constitute a relevant and challenging patient group that warrants interdisciplinary work-up and treatment. Several retrospective studies suggest that aggressive local treatment consisting of primary lung tumor resection and/or brain metastasis resection in patients with either solitary brain metastasis, 2 or 3 brain metastases can increase survival [14, 15]. However, for these patients tissue markers predicting OS and PFS are lacking and the role of TTF-1 in these patients is not well established. So far only one study published in 2023 on 137 patients with NSCLC/ADC brain metastases assessed the role of TTF-1, which was exclusively based on lung tissue biopsies at the time of diagnosed lung cancer [8]. TTF-1 + patients showed a median OS of 7 months [95% CI 5.91–8.09], whereas TTF-1- patients showed a median OS of 5.8 months [95% CI 4.1–7.5] (p = 0.024) and TTF-1 status was independently associated with outcome (p = 0.0034). Importantly, all these patients underwent whole brain radiation therapy and none of these patients underwent brain metastasis resection or SRS [8]. In our study, we focused instead on TTF-1 based on resected brain metastasis tissue from patients with treatment-naïve synchronous LUAD brain metastases. We could show that a negative brain metastasis tissue-specific TTF-1 status is associated with increased tumor cell proliferation on histopathological examination and tumor volume of brain metastases in pre-operative cMRI and that TTF-1 negativity was an independent marker for worse OS as well as intra- and extracranial PFS in LUAD brain metastasis. This is in line with previous data linking TTF-1 positivity to a more favorable prognosis, probably resulting from TTF-1’s role in maintaining the differentiated state of lung adenocarcinoma cells, suggesting a less aggressive tumor phenotype [13, 19]. TTF-1 expression was previously shown to be associated with decreased cell proliferation via repression of Ki67 expression [19]. Additionally, one study observed inverse correlation between TTF-1 immunohistochemical and Ki67 staining in 65 cases of NSCLC and 16 cases of SCLC which aligns with our observations in LUAD brain metastases [20].

Further, TTF-1 status seems to be an important factor in determining the risk of intracranial progression in patients with TTF-1 + patients tend to have a reduced risk of both extracranial and intracranial progression, with the reduction being more statistically significant for intracranial progression. In contrast, TTF-1- patients, exhibit an increased risk for both progression types, with the risk being notably higher for intracranial progression.

Intriguingly, we observed an inverse correlation between TTF-1 status and tumor edema, while tumor volume and edema volume of the dominating brain metastasis as such did not correlate significantly with each other. The latter aspect was previously observed in brain and melanoma brain metastases [21]. In this regard, Berghoff et al. showed that in newly diagnosed, treatment-naïve brain metastases edema volume of brain metastasis correlates with the density of infiltration of tumor-infiltrating lymphocytes (TILs), where TIL density correlated with OS [22]. In this study, we do not provide data concerning potential causes that explain our observation and whether TTF-1 expression can, to a part, explain edema differences, yet it is tempting to speculate that TTF-1 may also be associated with a different tumor microenvironment (TME) in LUAD brain metastases which may relate to the observed differences in size of perifocal edema. Interestingly, TTF-1- patients showed lower PD-L1 TPS scores in brain metastasis tissue compared to TTF-1 + patients which might be linked to a colder immune TME in TTF-1- brain metastases [23]. Our data showed no marked differences between TTF-1 + and TTF-1- patients regarding the anatomical site of brain metastases, brain metastasis burden, and prevalence of extracranial metastasis, nor was there a correlation between TTF-1 status and these parameters suggesting that TTF-1 expression as such may not be associated with metastatic tropism or metastatic spread patterns within the brain. As the exact role in regulating cell differentiation and proliferation and other processes specifically in brain metastases remain unexplored, precise, genomic studies in the context of well-annotated clinical cohorts such as recently published data from Skakodub et al. would be needed to assess differences between TTF-1- and TTF-1 + patients[24]. It is important to discuss the limitations of our study, including its retrospective nature, sample size, the absence of a central radiologic review and regular post-operative cMRI, and the lack of correlation of TTF-1 status with molecular data. In addition, we do not further distinguish into different types of postsurgical systemic therapies. Future prospective studies are necessary to validate these findings, and to perform more mechanistic experiments including studies on the role of TTF-1 and the TME. Correlation with molecular pathology-related data would be further important for a more precise molecular classification of TTF-1- patients. Lastly, we did not compare TTF-1 status between intracranial lesions, given the small cohort size of patients with two-stage resection. However, it would be interesting to evaluate different compartments (e.g. brain metastasis tissue 1 and 2 and or matched extracranial tissue) in terms of heterogeneity of TTF-1 expression. Finally, these studies could help to potentially incorporate TTF-1 status into patient risk stratification either as a prognostic or predictive marker for this important patient cohort of newly diagnosed LUAD brain metastasis patients.

## Conclusion

In conclusion, positive TTF-1 status in NSCLC adenocarcinoma patients with brain metastasis is associated with improved survival outcomes. This finding suggests the potential utility of TTF-1 as a prognostic biomarker in newly diagnosed LUAD brain metastasis, which could inform treatment decisions and guide the development of targeted therapies. Future research should aim to explore the biological mechanisms underlying the relationship between TTF-1 expression and tumor behavior, especially in the context of brain metastases. Understanding these mechanisms may lead to novel therapeutic approaches that exploit the molecular pathways associated with TTF-1 to improve outcomes for these patients.

## Supplementary Information

Below is the link to the electronic supplementary material.

Supplementary file1 (PDF 451 KB)

## Data Availability

Raw data will be made available on reasonable request via the corresponding authors. The R code will be accessible upon request via Github: https://github.com/dasilew/clinicalNSCLC_1.

## Abbreviations

ALK: Anaplastic lymphoma kinase
CI: Confidence interval
cMRI: Cranial magnetic resonance imaging
CT: Computed tomography
ecPFS: Extracranial progression-free survival
EGFR: Epidermal growth factor receptor
FFPE: Formalin-fixed paraffin-embedded
GPA: Graded prognostic assessment
HR: Hazard ratio
IHC: Immunohistochemistry
iRANO: Immunotherapy response assessment in neuro-oncology
icPFS: Intracranial progression-free survival
ICI: Immune checkpoint inhibition
LUAD: Lung adenocarcinoma
M1b: Stage IV disease with single extrathoracic metastasis (i.e. with solitary brain metastasis)
M1c: Stage IV disease with multiple extrathoracic metastases
NSCLC: Non-small cell lung cancer
OS: Overall survival
PFS: Progression-free survival
RECIST: Response evaluation criteria in solid tumors
ROS1: ROS proto-oncogene 1
SMIs: Small molecule inhibitors
SRS: Stereotactic radiosurgery
TME: Tumor microenvironment
TPS: Tumor proportion score
TTF-1: Thyroid transcription factor-1

## Declarations

### Previous presentations

Portions of this work were presented at the annual meeting of the European Association of Neurosurgical Societies (EANS), Sofia, Bulgaria, October 13–17, 2024.
