# Supplementary material for: TTF-1 negativity in synchronous M1b/M1c wildtype lung adenocarcinoma brain metastases predicts worse survival with increased risk of intracranial progression
Source: J Neurooncol. 2024 Dec 4;171(3):637–49. doi: 10.1007/s11060-024-04885-y (PMC11729080; doi:10.1007/s11060-024-04885-y)
Supplement: Supplementary file 1 — Supplementary file1 (PDF 451 KB) [file 11060_2024_4885_MOESM1_ESM.pdf]

Supplementary material

TTF-1 Negativity in Synchronous M1b/M1c Wildtype Lung Adenocarcinoma Brain Metastases Predicts Worse Survival with Increased Risk of Intracranial Progression

David Wasilewski[1, 2, 3*] Tommaso Araceli[4, 5*], Philip Bischoff[3, 6, 7], Anton Früh[1, 6], Rober Ates[1], Selin Murad[1], Niklas Jung[1], Jan Bukatz[1], Majd Samman[1, 8], Katharina Faust[1, 6], Julia Jünger[9], Martin Witzenrath[10], David Horst[3, 6, 7], Atik Baborie[11], Arend Koch[9], David Capper [3, 9], Frank L. Heppner[3, 9], Nils Ole Schmidt[4, 5], Peter Vajkoczy[1, 2, 6], Helena Radbruch[9], Markus J. Riemenschneider[11], Martin Proescholdt[4, 5], Julia Onken[1, 2, 3, 6$], Nikolaj Frost[2, 10$]

[1]Charité – Universitätsmedizin Berlin, Corporate Member of Freie Universität Berlin and Humboldt-Universität zu Berlin, Department of Neurosurgery, Berlin, Germany.

[2]Charité – Universitätsmedizin Berlin, Corporate Member of Freie Universität Berlin and Humboldt-Universität zu Berlin, Charité Comprehensive Cancer Center, Berlin, Germany.

[3]German Cancer Consortium (DKTK), partner site Berlin, and German Cancer Research Center (DKFZ), Heidelberg, Germany

[4]Department of Neurosurgery, University Regensburg Medical Center, Regensburg, Germany

[5]Wilhelm-Sander Neuro-Oncology Unit, University Regensburg Medical Center, Regensburg, Germany

[6]Berlin Institute of Health at Charité – Universitätsmedizin Berlin, Berlin Institute of Health (BIH) Charité, Charitéplatz 1, 10117 Berlin, Germany

[7]Charité – Universitätsmedizin Berlin, Corporate Member of Freie Universität Berlin and Humboldt-Universität zu Berlin, Institute of Pathology, Berlin, Germany.

[8]King Salman Medical City, Neurosience Institute, Department of Neurosurgery, Medina, Saudi Arabia.

[9]Charité – Universitätsmedizin Berlin, Corporate Member of Freie Universität Berlin and Humboldt-Universität zu Berlin, Institute of Neuropathology, Berlin, Germany.

[10]Charité – Universitätsmedizin Berlin, Corporate Member of Freie Universität Berlin and Humboldt-Universität zu Berlin, Department of Infectious Diseases and Pulmonary Medicine, Berlin, Germany.

[11]Department of Neuropathology, University Regensburg Medical Center, Regensburg, Germany

* Authors share first authorship

$ Authors share last authorship

Corresponding author:
Dr. med. David Wasilewski, MD
Charité – Universitätsmedizin Berlin, Corporate Member of Freie Universität Berlin and Humboldt-Universität zu Berlin, Department of Neurosurgery, Berlin, Germany.
Email: david.wasilewski@charite.de

´

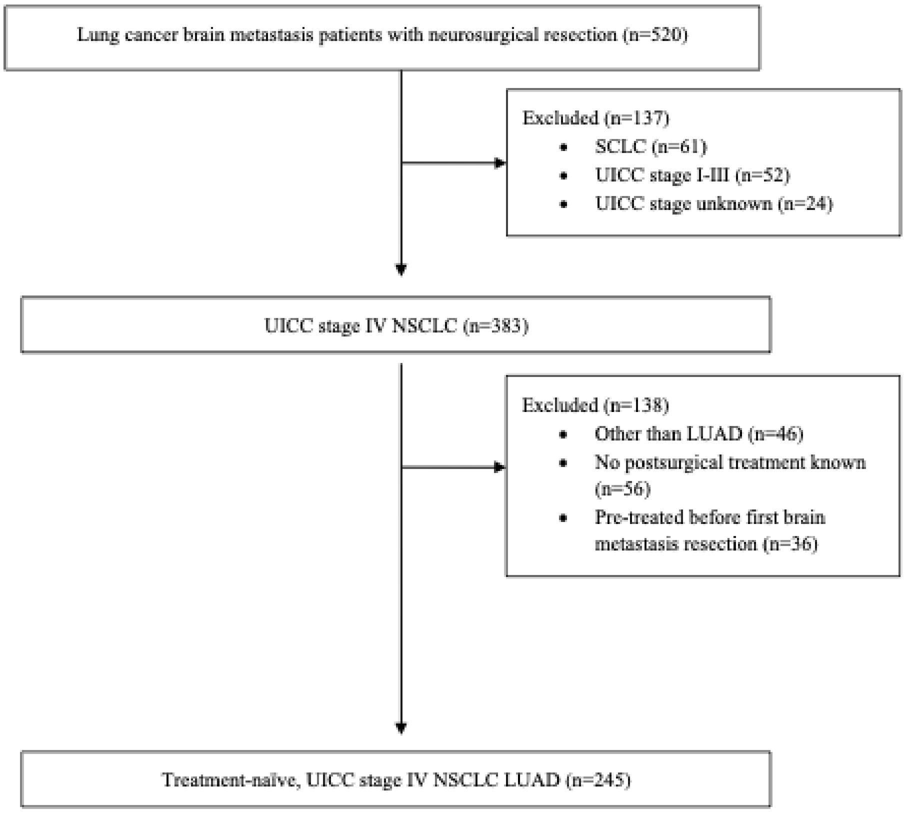

Supplementary figure 1: CONSORT diagram

A CONSORT diagram representing the entire study population. Our cohort involved 245 patients who underwent craniotomy and microsurgical brain metastasis resection and had evidence of NSCLC/ADC on analysis of brain metastasis tissue without evidence of prior local or systemic treatments before brain metastasis resection.

Supp. Figure 2

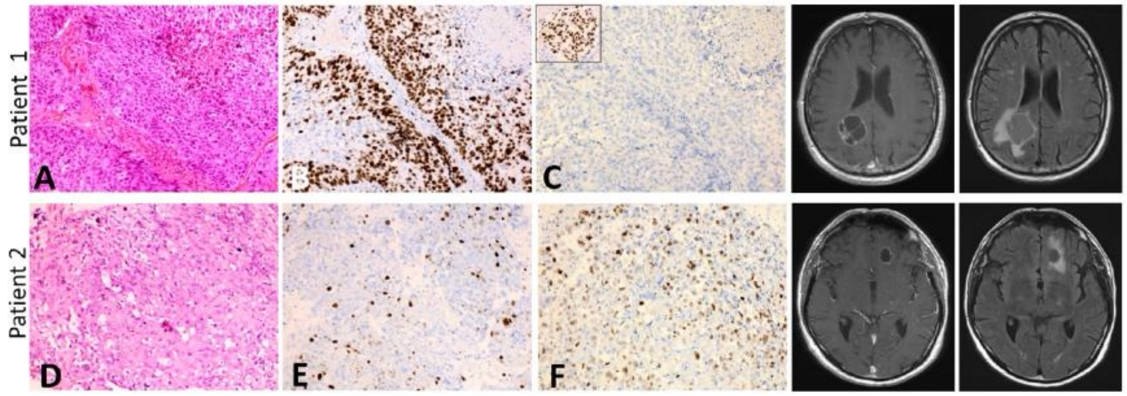

Supplementary figure 2: Representative images of TTF-1 and Ki67 IHC staining grouped according to TTF-1 status

Comparison of TTF-1- (patient 1, top row, with focal necrosis at the top right corner) and TTF-1+ (patient 2, bottom row) LUAD brain metastasis. H&E (**3A, 3F**), Ki67/MiB1 (**3B, 3G**) and TTF-1 (**3C, 3H**), contrast-enhance T1-weighted cMRI (**3D, 3I**) and T2-weighted FLAIR imaging (**3E, 3J**) (Magnification of all images x100. Inset in C is the TTF-1 positive control.

Supp. Figure 3

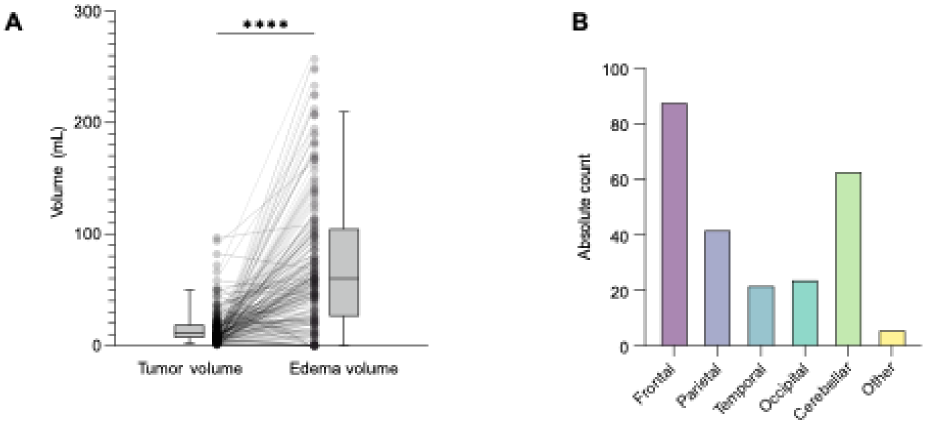

Supplementary figure 3: Descriptive analysis of anatomical parameters in the total patient cohort

Descriptive statistics of clinico-pathological parameters of the whole patient cohort including volume of the dominant brain metastasis the edema volume of the dominant brain metastasis, the relationship of paired tumor volume and edema volume (assessed via a matched-pairs Wilcoxon signed rank test) (**4A**) and a bar graph depicting the absolute count of metastases across various brain regions: frontal, parietal, temporal, occipital, cerebellar, and others (e.g. insular region, lesions associated with the third ventricle, lesions of the skull base (**4B**).

Supp. Figure 4

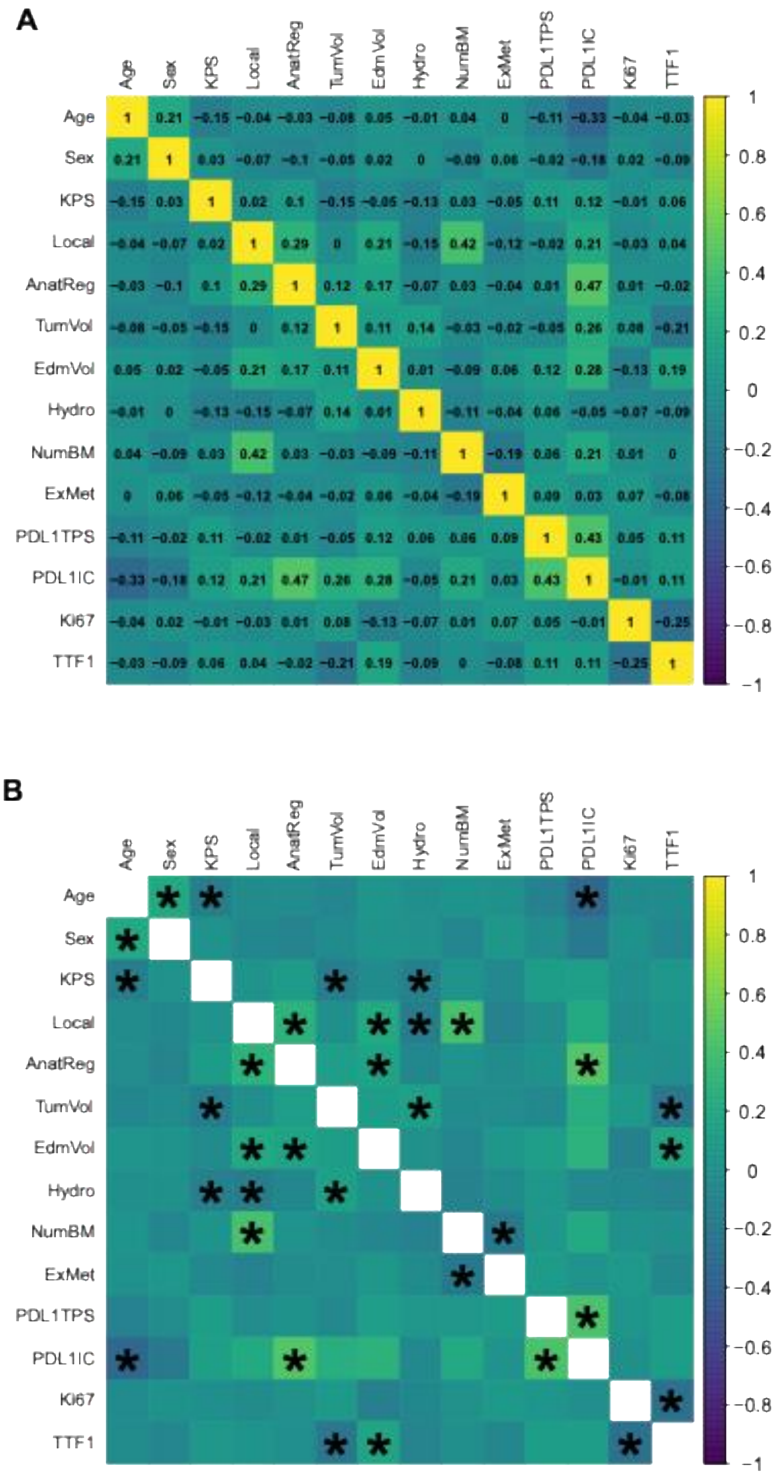

Supplementary figure 4: Comparing tumor and edema volumes between TTF-1 negative (TTF1-) and TTF-1 positive (TTF1+) groups

Supplementary Figure 4 illustrates the distribution and comparison of tumor and edema volumes stratified by TTF-1 status, presented in four pairs of plots. Panels A-D show analyses related to tumor volume: A and B display bar and box plots with tumor volume data that include outliers. C and D present similar plots where outliers have been excluded using Tukey's fences method (1.5 × IQR). Panels E-H depict analyses for edema volume: E and F feature bar and box plots including outliers. G and H show corresponding plots where outliers were excluded using the same statistical method. These visualizations provide a comprehensive view of how tumor and edema volumes differ based on TTF-1 status, both with and without the inclusion of outliers.

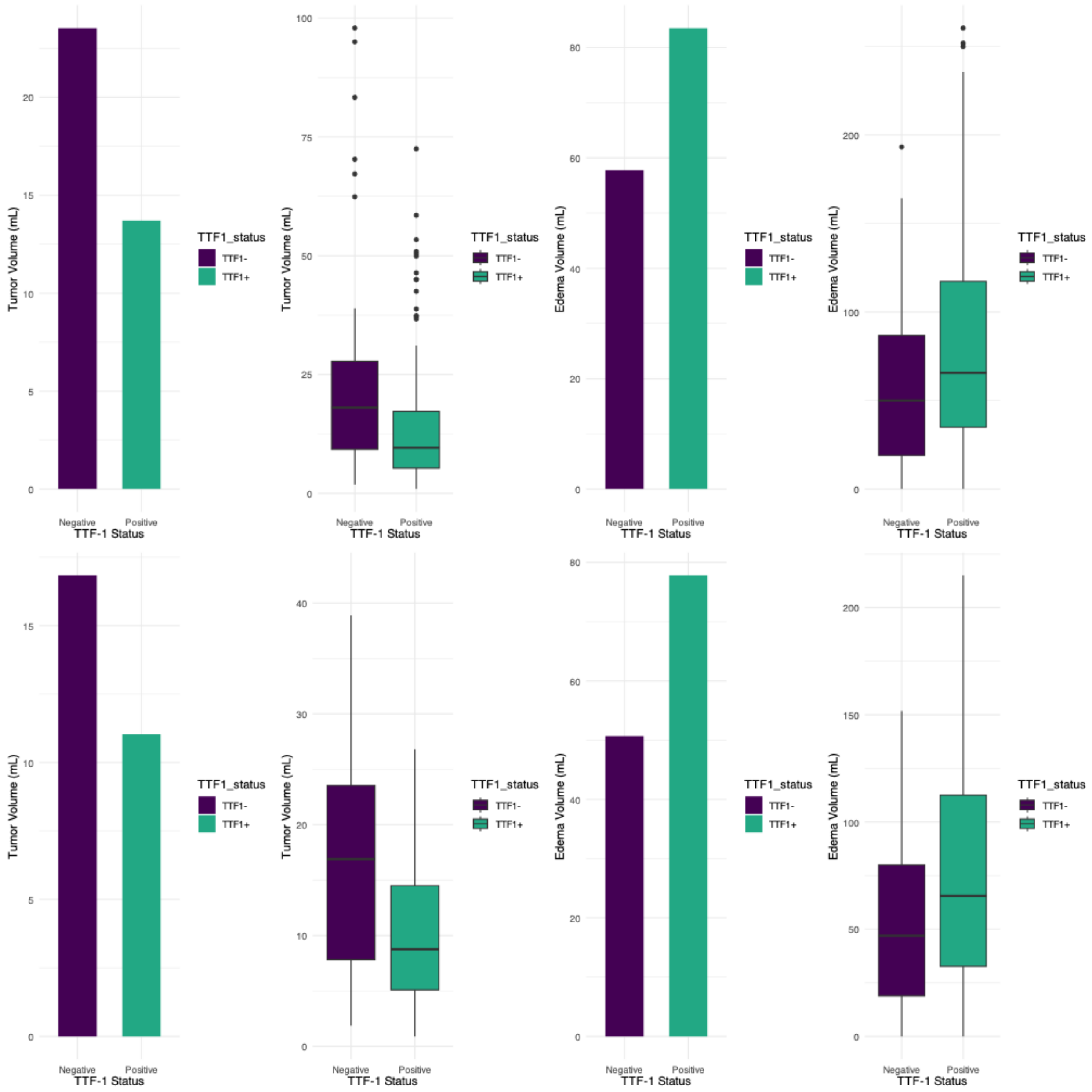

Supplementary figure 5: Exploratory correlation analysis of TTF-1 status with clinico-pathological covariates

Correlation between TTF-1 status and clinico-pathological parameters such as brain metastasis burden, number of brain metastasis and localization of dominant brain metastasis, tumor or edema volume and Ki67 index were plotted using a correlation plot based on a Pearson correlation analysis (**5A**). A matrix of p-values indicating corresponding significance levels to the correlation, where significant correlations are marked with an asterisk (**5B**) Abbreviations used

in these correlation plots: Age: Age of patients in years, Sex: Gender of patients (Male/Female), KPS: Karnofsky Performance Status, a standard measure of patients' ability to perform ordinary tasks, ds GPA: diagnosis-specific Graded Prognostic Assessment, a tool used to predict outcomes in brain metastasis patients, Local: Location of tumor within the brain, AnatReg: anatomical region affected by the dominant brain metastasis, TumVol: tumor volume of the dominant brain metastasis (mL), EdmVol: edema volume of the dominant brain metastasis (mL), Hydro: Hydrocephalus, indicating the presence or absence of fluid accumulation in the brain; NumBM: number of brain metastases (brain metastasis burden), ExMet: extracranial metastases, indicating the presence or absence of cancer spread outside the brain at time of brain metastasis resection, PDL1TPS: brain metastasis-specific PD-L1 tumor proportion score, PDL1IC: brain metastasis-specific PD-L1 immune cell score, Ki67: brain metastasis-specific Ki-67 index, TTF-1: brain metastasis-specific Thyroid Transcription Factor 1 (TTF-1).

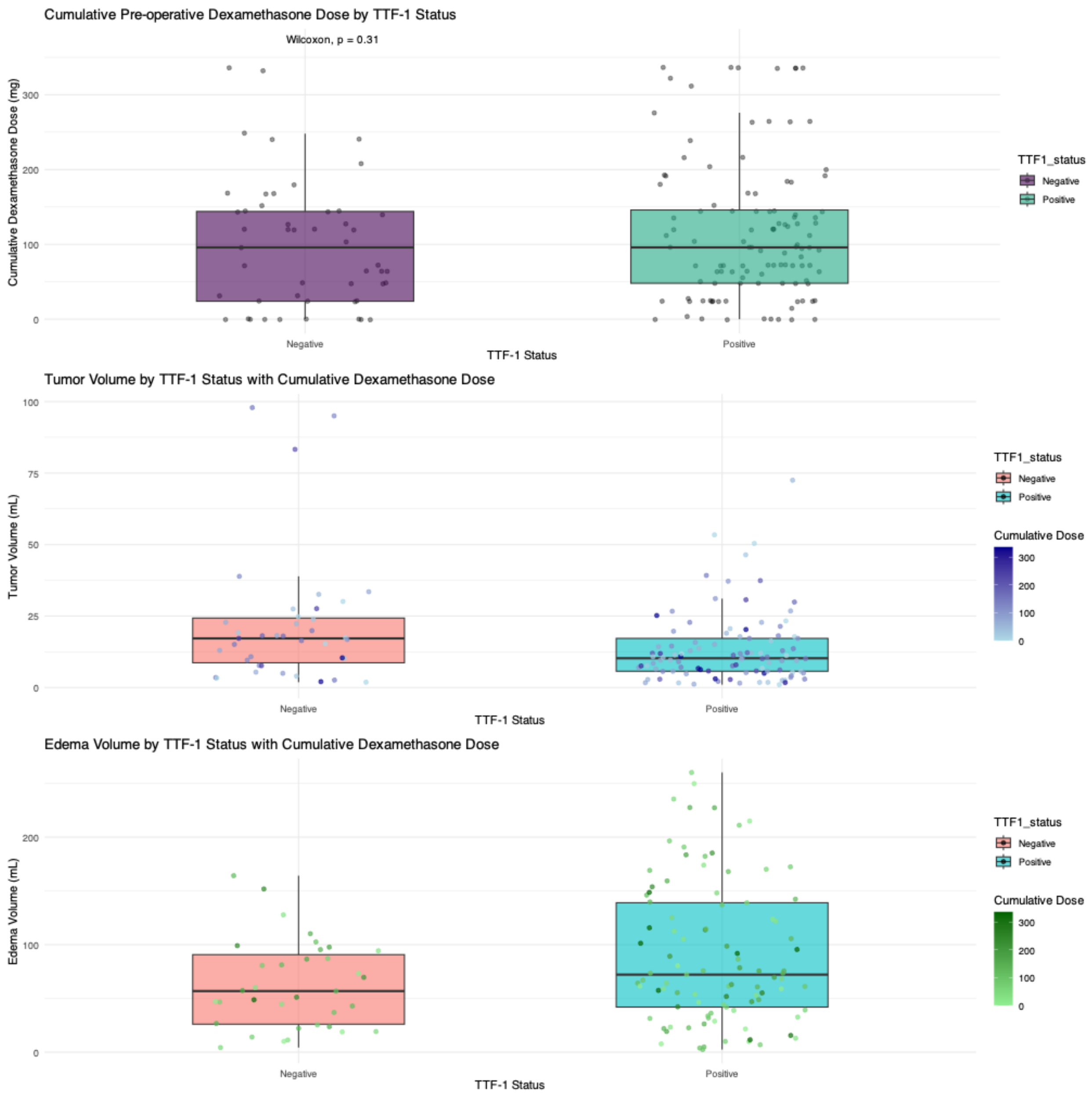

Supplementary figure 6: Relationship of tumor and edema volumes and cumulative pre-operative dexamethasone group in TTF-1+ and TTF-1 patients

Bar plots depicting the pre-operative cumulative dexamethasone in TTF-1- and TTF-1+ patients (**6A**). Box plot visualizing tumor volume by TTF-1 status in relation to pre-operative cumulative dexamethasone dose (**6B**), and edema volume by TTF-1 status in relation to pre-operative cumulative dexamethasone dose (**6C**).
